# A monomethyl auristatin E-conjugated antibody to guanylyl cyclase C is cytotoxic to target-expressing cells *in vitro* and *in vivo*

**DOI:** 10.1371/journal.pone.0191046

**Published:** 2018-01-25

**Authors:** Melissa Gallery, Julie Zhang, Daniel P. Bradley, Pamela Brauer, Donna Cvet, Jose Estevam, Hadi Danaee, Edward Greenfield, Ping Li, Mark Manfredi, Huay-Keng Loke, Claudia Rabino, Brad Stringer, Mark Williamson, Tim Wyant, Johnny Yang, Qing Zhu, Adnan Abu-Yousif, O. Petter Veiby

**Affiliations:** 1 Molecular & Cellular Oncology, Millennium Pharmaceuticals, Inc., a wholly owned subsidiary of Takeda Pharmaceutical Company Limited, Cambridge, MA, United States of America; 2 Cancer Pharmacology, Millennium Pharmaceuticals, Inc., a wholly owned subsidiary of Takeda Pharmaceutical Company Limited, Cambridge, MA, United States of America; 3 Biomedical Imaging, Millennium Pharmaceuticals, Inc., a wholly owned subsidiary of Takeda Pharmaceutical Company Limited, Cambridge, MA, United States of America; 4 Protein Sciences, Millennium Pharmaceuticals, Inc., a wholly owned subsidiary of Takeda Pharmaceutical Company Limited, Cambridge, MA, United States of America; 5 Biomarker Assay & Exploratory Biology, Millennium Pharmaceuticals, Inc., a wholly owned subsidiary of Takeda Pharmaceutical Company Limited, Cambridge, MA, United States of America; 6 Oncology Biochemistry, Millennium Pharmaceuticals, Inc., a wholly owned subsidiary of Takeda Pharmaceutical Company Limited, Cambridge, MA, United States of America; 7 Molecular Pathology, Millennium Pharmaceuticals, Inc., a wholly owned subsidiary of Takeda Pharmaceutical Company Limited, Cambridge, MA, United States of America; 8 US Medical Affairs, Millennium Pharmaceuticals, Inc., a wholly owned subsidiary of Takeda Pharmaceutical Company Limited, Cambridge, MA, United States of America; 9 Translational Medicine, Millennium Pharmaceuticals, Inc., a wholly owned subsidiary of Takeda Pharmaceutical Company Limited, Cambridge, MA, United States of America; 10 DMPK, Millennium Pharmaceuticals, Inc., a wholly owned subsidiary of Takeda Pharmaceutical Company Limited, Cambridge, MA, United States of America; 11 Global Biotherapeutics, Millennium Pharmaceuticals, Inc., a wholly owned subsidiary of Takeda Pharmaceutical Company Limited, Cambridge, MA, United States of America; Mie University Graduate School of Medicine, JAPAN

## Abstract

Guanylyl cyclase C (GCC) is a cell-surface protein that is expressed by normal intestinal epithelial cells, more than 95% of metastatic colorectal cancers (mCRC), and the majority of gastric and pancreatic cancers. Due to strict apical localization, systemically delivered GCC-targeting agents should not reach GCC in normal intestinal tissue, while accessing antigen in tumor. We generated an investigational antibody-drug conjugate (TAK-264, formerly MLN0264) comprising a fully human anti-GCC monoclonal antibody conjugated to monomethyl auristatin E via a protease-cleavable peptide linker. TAK-264 specifically bound, was internalized by, and killed GCC-expressing cells *in vitro* in an antigen-density-dependent manner. In GCC-expressing xenograft models with similar GCC expression levels/patterns observed in human mCRC samples, TAK-264 induced cell death, leading to tumor regressions and long-term tumor growth inhibition. TAK-264 antitumor activity was generally antigen-density-dependent, although some GCC-expressing tumors were refractory to TAK-264-targeted high local concentrations of payload. These data support further evaluation of TAK-264 in the treatment of GCC-expressing tumors.

## Introduction

Gastrointestinal cancers are among the most common cancers in the United States, with an estimated 291,150 new cases of digestive system cancers in 2015; notably, colon/rectum and pancreatic cancers are the third and fourth most common causes of cancer-related deaths, with an estimated 49,700 and 40,560 deaths, respectively, in 2015 [1]. Prognosis is poor with many gastrointestinal malignancies; for example, approximately one fifth of patients with colorectal cancer (CRC) have distant metastases [2–5]. Current chemotherapeutic options for metastatic CRC (mCRC) include combination chemotherapies and molecularly targeted monoclonal antibody therapies [6]; however, although treatment options are expanding, 5-year survival for patients with distant metastases is only approximately 10% [5–7]. Similarly, median survival for patients with pancreatic cancer is only 4–6 months [8]. Clearly, investigations into new treatment strategies are warranted.

Guanylyl cyclase C (GCC) is a transmembrane cell surface receptor that functions in the maintenance of intestinal fluid, electrolyte homeostasis, and restriction of cell proliferation [9]. In normal human tissues, GCC expression is restricted to the mucosal cells lining the small intestine, large intestine, and rectum [10, 11]. GCC expression is maintained upon neoplastic transformation of intestinal epithelial cells, with expression in >95% of primary and metastatic colorectal tumors [10, 12–14] and in 60–70% of gastric, esophageal, and pancreatic cancers [15–17]. The tissue-restricted expression and consistent association with CRC has been exploited for use of GCC as a diagnostic and prognosis marker for this disease [10, 14, 18–20]. Recent studies have suggested GCC expression is a marker of therapeutic response in mCRC [11].

In normal intestinal tissue, GCC is expressed on the apical side of epithelial cell tight junctions that form an impermeable barrier between the luminal environment and vascular compartment [21, 22]. As such, systemic intravenous administration of a therapeutic anti-GCC antibody should not affect normal intestinal GCC receptors, while having access to extraintestinal GCC-expressing tumors. The selective targeting of colon tumor cells with GCC ligands has been demonstrated *in vivo* [23, 24]. In one study, a radionuclide-conjugated GCC ligand was shown to selectively target GCC-expressing colon tumor xenografts in mice, with no accumulation in the normal mouse intestinal epithelium [24]. Similarly, formation of metastases was significantly reduced in mouse models of mCRC through immunization with GCC-expressing viral vectors with no evidence of autoimmunity [25]. Additionally, GCC internalizes through receptor-mediated endocytosis upon ligand binding, making it a candidate for intracellular delivery of anticancer therapeutic proteins [14, 26].

Antibody-drug conjugates (ADCs) are an emerging therapeutic modality for the targeted delivery of potent cytotoxic agents to tumors [27, 28]. Recent developments have included the anti-CD30 ADC brentuximab vedotin [29, 30], which is approved in the USA and EU for the treatment of relapsed or refractory Hodgkin lymphoma and systemic anaplastic large cell lymphoma, and the human epidermal growth factor receptor 2 (HER2)-targeted ADC trastuzumab emtansine [31], which is approved in the USA and EU for the treatment of HER2-positive breast cancer. Many other ADCs are currently in clinical development, building upon these successes [32]; the attributes of GCC make it an enticing target for an ADC strategy.

TAK-264 (formerly MLN0264) is a novel ADC consisting of a fully human anti-GCC monoclonal antibody conjugated to the potent microtubule-disrupting agent monomethyl auristatin E (MMAE) via the protease-cleavable peptide maleimido-caproyl-valine-citrulline [vc]. MMAE and the linker technology are licensed from Seattle Genetics, Inc. In the present study, we demonstrate that TAK-264 binds to, is internalized by, and leads to dose-dependent, selective cell death and antitumor activity in GCC-expressing cells *in vitro* and *in vivo*, respectively. Thus, TAK-264 has potential for development as an ADC therapeutic in GCC-expressing tumors.

## Materials and methods

### Generation of GCC protein and anti-GCC antibodies

Recombinant protein containing the extracellular domain of human GCC fused to human immunoglobulin G (IgG1) Fc was used to immunize female C57BL/6 mice (Taconic Farms, Germantown, NY) or Xenomice (Abgenix, Fremont, CA), for the generation of hybridomas. Monoclonal antibodies were generated by fusing spleen cells and SP 2/0 myeloma cells (ATCC No. CRL8-006, Rockville, MD) with a polyethylene glycol 1450 (ATCC) fusion procedure [33]. Hybridoma supernatants were screened for anti-GCC antibodies using indirect ELISA and flow cytometry on GCC transfected HT-29 cells. Positive hybridomas were subcloned to achieve monoclonality.

Recombinant anti-GCC monoclonal antibody was prepared using molecular biology techniques to isolate the coding sequences for the variable regions from the hybridoma cell line and combine them with sequences for human IgG1 and human kappa constant regions in an in-house-generated expression vector, pTOK58D. CHO DG44 cells adapted to serum-free, suspension media were transfected with this expression construct using electroporation, selected with Geneticin (G418), amplified with methotrexate (MTX), and limited-dilution cloned to generate a high-producing cell line to support pre-clinical and phase 1 clinical studies. This cell line was shown to be stable, mycoplasma free, and bio-burden free, and was used for production of the anti-GCC monoclonal antibody, which was used for conjugation to MMAE to generate TAK-264.

For large-scale production of the unconjugated anti-GCC monoclonal antibody, the above-generated cell line was scaled up in serum-free, animal-component-free medium in bioreactors or biowave bags, fed every 3 days, and harvested when the viability was <40%. The supernatant underwent standard Protein A capture purification, concentration/diafiltration, and a final endotoxin cleanup using detoxigel. Concentration of the anti-GCC monoclonal antibody was determined using the formula (A_280_–A_320_)/1.4, and endotoxin content was determined using the Pyrochrome^®^ chromogenic test kit (Associates of Cape Cod). For conjugation purposes, the antibody was concentrated to 10 mg/mL, using Vivaspin 15 MWCO 30,000 kDa. The anti-GCC monoclonal antibody was conjugated to MMAE by Seattle Genetics according to previously published protocols. An average of 3.7 MMAE molecules were conjugated per antibody.

### Quantitative RT-PCR analysis of GCC in human tissues

Frozen human tissues for mRNA extraction were procured from ProteoGenex, Inc. (Culver City, CA, USA). Semi-quantitative reverse transcription polymerase chain reaction (RT-PCR) was undertaken using TaqMan^®^ probe specific for GCC. Relative expression levels were normalized according to the expression of beta 2 microglobulin.

### Cell lines

All cell lines used in this study were generated internally at Millennium Pharmaceuticals, Inc., a wholly owned subsidiary of Takeda Pharmaceutical Company Limited. HEK293 cells (ATCC, Manassas, VA, USA) were maintained in 90% Dulbecco’s Modified Eagle’s Medium (DMEM, 11995–040, Invitrogen), 10% fetal bovine serum (FBS, SH 30071.02, Hyclone), 1% L-glutamine (25030–081, Invitrogen), and (HEK293-GCC cells) 10 μg/mL of blasticidin (R21001, Invitrogen). All cells were grown at 37°C in a humidified atmosphere with 5% CO_2_. The HEK293-vector cells were generated by using a vector named pN8ΦβmycSV40 that was created by Millennium Pharmaceuticals. The HEK293-GCC cells were generated by cloning in the full-length GCC sequence into the same vector pN8ΦβmycSV40. After confirmation by sequencing of pN8ΦβmycSV40 human GCC DNA, the stable cell lines were generated by transfection with Lipofectamine 2000. Cells (2 × 10^6^) were transfected with 24 μg of DNA + 60 μL of Lipofectamine 2000 in 1500 μL of Opti-MEM each. The transfected cells were maintained in complete media for 48 h before changing to selection media for 2 weeks. Western blot analysis using anti-mouse GCC(3G1), as well as FACS analysis with anti-human GCC, confirmed the generation of stable cell lines expressing the control vectors or human GCC.

Cryopreserved early-passage cells were re-established in culture for all experiments. Transcriptional profiling of cell lines confirmed that vector control cell lines differed only in the expression level of GCC.

### Western blot analysis

Cells were collected and washed in Dulbecco’s phosphate buffered saline (PBS) without CaCl_2_ and without MgCl_2_ (14190–144, Invitrogen) and spun down. For denaturing polyacrylamide gels, cell pellets were lysed in buffer containing 10 mM Tris (pH 7.5) with 1% Triton X-100, supplemented with protease inhibitor (complete-mini tablets, Roche), by three rounds of vortexing, and incubation on ice for 5 min. Lysates were cleared by centrifugation and the protein concentrations in the supernatants were determined using the Bradford assay. Equal amounts of total protein (50 μg) were denatured and loaded on 7% Tris-Acetate SDS-PAGE gels (EA 0358, Invitrogen), transferred to nitrocellulose membrane (Invitrogen), using Invitrogen’s Tris-Acetate transfer buffer and XCell Blot Module for 1.5 h at 30 V. Membranes were blocked with 5% nonfat skim milk in Tris-buffered saline-Tween 20 (TBS-T) for 1 h at room temperature, washed with TBS-T and incubated for 3 h with primary antibodies (3G1 or an anti-actin goat polyclonal antibody [Santa Cruz Biotechnology]). After washing with TBS-T, membranes were incubated for 1 h with secondary antibodies (HRP-conjugated goat anti-mouse IgG [Perkin-Elmer] and donkey anti-goat IgG [Santa-Cruz Biotechnology], respectively). Blots were developed using enhanced chemiluminescence (Amersham, Piscataway, NJ).

### Cell surface binding assay

Antibody binding to cells was evaluated by an indirect immunofluorescence assay using flow cytometry. 1 × 10^6^ cells/well were plated in a V-bottom 96-well plate and incubated on ice for 1 h with serial antibody dilutions of 1–0.001 μg/mL. Cells were washed twice with 3% FBS in ice-cold PBS and incubated with 1:200 mouse anti-human PE IgG (Southern Biotech 2043–09) for 1 h on ice. Cells were washed again and analyzed by flow cytometry on a Becton Dickinson FACScan flow cytometer. Data was analyzed using Winlist 4.0 Software (Verity Software House, Topsham, ME) and background-corrected mean fluorescence intensity was determined. Mean fluorescence intensities were plotted versus antibody concentration using Winlist 4.0 Software (Verity Software House, Topsham, ME).

### Antibodies for Western blot analysis and immunofluorescence

The anti-actin goat polyclonal (SC-1616) and anti-goat IgG (donkey) HRP secondary (SC-2056) were obtained from Santa Cruz Biotechnology. The anti-mouse IgG (goat) HRP-labeled secondary (NEF 822) was purchased from Perkin Elmer. For immunofluorescence, anti-GCC monoclonal antibodies were used as primaries and Alexa-fluor conjugated goat anti-human antibodies (Invitrogen) were used as secondary antibodies.

### Internalization assay

Cells were grown on coverslips and placed on ice for 10 min prior to adding antibody (TAK-264 10 μg/mL) in cold culture medium. Cells were maintained on ice for 20 min to allow cell surface binding by the antibody. For internalization, antibody-containing medium was replaced with fresh culture medium and cells were incubated at 37°C for 3 h. Control plates were kept on ice for the same length of time. Cells were briefly rinsed in PBS and fixed for 5 min in 4% paraformaldehyde at room temperature. Cells were subsequently washed in PBS, permeabilized for 15 min in 0.5% Triton X-100, and incubated in 0.5% blocking reagent (Roche, cat. #1095176) in 100 mM Tris-HCl pH 7.5, 150 mM NaCl. An appropriate dilution of Alexa Fluor^®^ 488 conjugated secondary antibody (ThermoFisher Scientific) was made in 0.5% blocking reagent and added to the cells for 30 min at room temperature. Cells were then washed three times for 5 min in PBS. Coverslips were mounted on glass slides using Vecta Shield Mounting Medium (Vector Laboratories). Staining was visualized by laser scanning confocal microscopy (Zeiss Pascal) and analyzed using Axiovert software.

### Determination of MMAE concentration following TAK-264 *in vitro* and *in vivo*

For *in vitro* assessment, 4 million HEK293-GCC cells were seeded in complete media, into 10 cm^2^ plates, 48 h prior to performing the experiment. On the day of experiment, media was carefully removed and cells were washed with 2 mL ice-cold media without FBS. Media was aspirated and 9 mL ice-cold media was added to each plate. Plates were placed on ice for 10 min, followed by addition of 1 μg of ADC to each plate, and incubated at either 4°C or 37°C for the time indicated. For each condition, media was collected at the completion of the incubation time at 4°C or 37°C. Plates were washed with 1 mL PBS and scraped to remove adherent cells. Samples were collected and spun down in centrifuge tubes at 1000 rpm. Tubes containing cell culture supernatant and cell pellets were stored at -80°C, until analyzed.

MMAE in tumor samples was determined by extraction of the tumor and homogenizing in mouse plasma based on weight (1:4, equivalent weight in mg vs. plasma volume in μL) on a FastPrep-24 homogenizer (MP Biomedicals, Solon, OH) for concentration analysis. Quantification of MMAE was conducted using a method based on liquid chromatography–tandem mass spectrometry (LC/MS/MS) methodology. A protein-precipitation method was used to extract MMAE from the bio-matrix. The internal standard used for analysis was a stable isotope-labeled MMAE. The dynamic range of the assay was 0.02–50 ng/mL.

### *In vitro* cytotoxicity assay

HEK293-vector and HEK293-GCC cells were plated in 96-well plates at a cell density of 4000 cells/well and exposed to a titration of the unconjugated anti-GCC monoclonal antibody or TAK-264 for 96 h. Cell viability was assessed using the WST assay (Roche, Indianapolis, IN), according to manufacturer’s directions. Triplicate wells were used for each treatment, and viability was normalized to untreated cells. Error was calculated as the standard error of the mean (SEM) of three independent experiments.

### Human xenograft tumor studies

All animal research and veterinary care were performed at Takeda Boston, and the study was conducted using a protocol approved by the Takeda Boston Institutional Animal Care and Use Committee (IACUC) in a facility accredited by the Association for Assessment and Accreditation of Laboratory animal Care International (AAALAC). Immunocompromised mice were housed in a controlled environment and received food and water ad libitum.

Six- to seven-week old CB17 SCID female mice were inoculated subcutaneously with 5 × 10^6^ of HEK293-GCC cells or human primary tumors in DMEM without 10% FBS medium. For PHTX studies (PHTX-09C, PHTX-21C, PHTX-07C, PHTX-17C, and PHTX-11C), mice were implanted subcutaneously in the right flank with tumor fragments from primary human colon tumor tissue propagated in mice. Tumor growth was monitored using Vernier calipers, and the mean tumor volume was calculated using the formula (0.5 × [length × width^2^]).

For *in vivo* pharmacokinetic and pharmacodynamic studies, when the mean tumor volume reached approximately 500 mm^3^, animals were randomized into treatment groups (n = 3, TAK-264 treatment group; n = 4, vehicle group). Mice received a single intravenous dose of vehicle or TAK-264 1.875 or 3.75 mg/kg. The animals were sacrificed at defined time points (1, 4, 8, 24, and 48 h, and 4 days) following a single intravenous administration of TAK-264, and tumor and whole blood samples were harvested. The blood samples were transferred into serum separator tubes (BD#365956).

For *in vivo* activity studies, mice were randomized into treatment groups of 10 mice per group when tumors reached approximately 200 mm^3^. Mice were dosed intravenously (0.1 mL dosing volume) once-weekly for a total of three doses. The treatment groups included vehicle (0.9% saline), 209-vcMMAE (non-targeting control antibody) at 7.5 and 10 mg/kg, TAK-264 at 1.875, 3.75, 7.5, and 10 mg/kg, and free MMAE at 0.3 mg/kg (toxin equivalent). Tumor growth inhibition was calculated at last day with vehicle control group using the formula (control average tumor volume–treated average tumor volume) × 100 / (control average volume).

Mice were anesthetized using an isoflurane/oxygen mix and kept under anesthesia while tumor fragments were implanted, and no analgesics were used or required per IACUC guidelines. This method of anesthesia was used due to its short induction and recovery time and the reliability of its effects [34]. There were no unexpected animal deaths due to experimental procedures in the above-mentioned *in vivo* studies. Mice were euthanized using inhaled CO_2_ when the following humane endpoints were reached: tumor volumes reaching ≥10% of the mouse’s body weight, tumor length ≥2 cm, or tumors of any size interfering with eating, drinking, urinating, defecating, or walking. No mice showed any signs illness due tumor formation. Animal health observations were performed twice daily at 10 AM and 3 PM by our husbandry staff. Temperature and relative humidity within the barrier facility rooms were checked (VWR thermometers) and recorded every morning by husbandry staff. The temperature of the rooms was maintained at 70°F, and temperature fluctuations of ±2°F degrees were reported to the Animal Facility Supervisor. The relative humidity for animal holding rooms was set to 50%. Any reading more than ±20% was also immediately reported to the Animal Facility Supervisor. Animals were housed on Alpha Dri® or Alpha Dri+^®^ bedding with two forms of enrichment (one of which had to be for sheltering or nesting) in Thoren cage setups that had been autoclaved. Water bottles were filled with chlorinated RO water, capped with a neoprene stopper, and then autoclaved in a covered container before placing them on the wire lid of the Thoren cage. Mice were fed commercially irradiated PicoVac^®^ Rodent Diet 20 –Irradiated (#5062). Cage setups as well as bottle water and food were changed once a week (or whenever necessary). All rodent holding rooms were on a ‘12-h on’ (6 AM to 6 PM) and ‘12-h off’ (6 PM to 6 AM) cycle.

### Immunohistochemistry

Detection of phospho-histone H3 was performed on 5 μm-thick, deparaffinized xenograft tumor sections. Slides were incubated for 1 h at 37°C with anti-phospho-histone H3 antibody (Upstate 06–570; 40 μg/mL) diluted in Dako diluents (DAKO USA). Secondary goat anti-rabbit rhodamine-red-X conjugate (Jackson Immunoresearch 111-295-144; 30 μg/mL) was added for 30 min at room temperature. DAPI Vectashield^®^ HardSet™ medium (Vector Laboratories, Burlingame, CA) was used as a chromatin counterstain. Images were captured with a Nikon Eclipse E800 (20x objective) and analyzed with MetaMorph 6.3r7 software (Molecular Devices, Downingtown, PA).

GCC protein levels in formalin-fixed paraffin-embedded tissues were assessed on 5 μm-thick sections. Slides were incubated with rabbit monoclonal anti-human GCC antibody (Takeda Pharmaceuticals International Co.; 3.5 μg/mL), diluted in Dako diluents (DAKO USA), for 1 h on the Ventana Medical Systems (Tucson, AZ) Discovery XT^®^ automated stainer. Antibodies were biotinylated with a rabbit anti-goat secondary antibody (Vector Laboratories) and developed with the 3,3’-diaminobexidine (DAB) substrate map system (Ventana Medical Systems). Slides were counterstained with hematoxylin and imaged using the Aperio whole slide scanning system. Colon tissue microarrays (US Biomax) were stained and scored by two independent pathologists following a protocol developed at Qualtek Molecular Laboratories on their Tek-Mate automated stainer. Briefly, a composite scoring system (0, 1, 2, or 3) was established that captures both staining intensity (negative, weak, moderate, and strong) and the proportion of cells that exhibit the respective staining intensity. This information was used to generate a modified *H*-score that follows the following formula: *H*-score = (% membrane at 0 + % cytoplasm at 0) × 0 + (% membrane at 1 + % cytoplasm at 1) × 1 + (% membrane at 2 + % cytoplasm at 2) × 2 + (% membrane at 3 + % cytoplasm at 3) × 3. Thus, this score produces a continuous variable that ranges from 0 to 600. Bridging studies were performed to ensure consistency of the data.

### Statistical analysis

One-way ANOVA using the Dunnett’s multiple comparison test (GraphPad Prism 5 Software) identified time points with significance.

## Results

### GCC expression is restricted to normal and malignant intestinal epithelial cells

**Fig 1:**
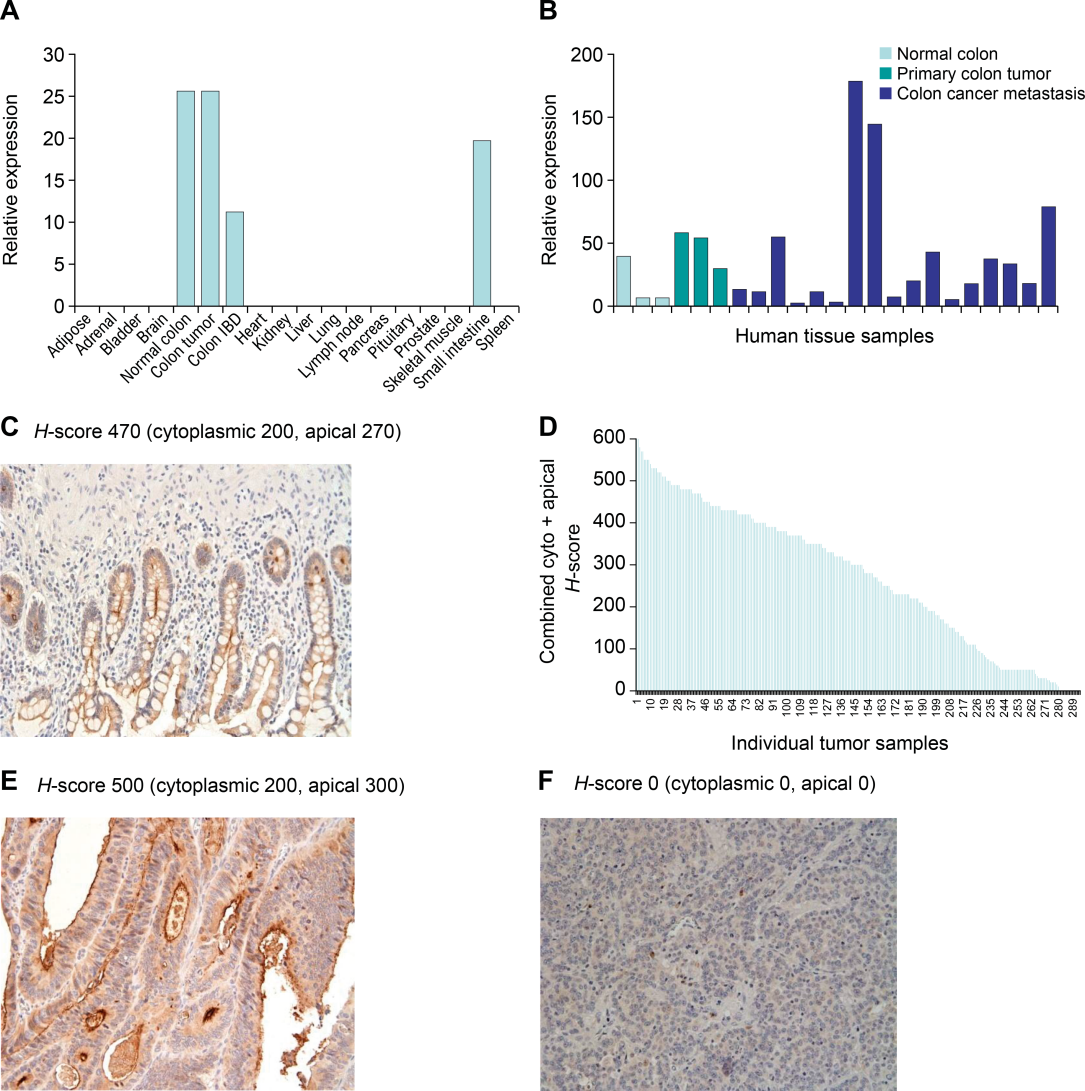
GCC expression analysis by quantitative RT-PCR and immunohistochemistry. GCC gene expression was analyzed in (A) a panel of human tissue samples and (B) normal human colon, primary and mCRC cancer samples. (C–F) Expression of GCC in human clinical samples was determined by immunohistochemistry. The intensity of GCC expression was determined by pathologist review and scored according to GCC intensity in the cytoplasm (cyto) and membrane-associated (apical) H-score from 0–300. (C) Expression of GCC in normal colon tissue. (D) A graphical presentation of the combined (cyto + apical) H-score of >300 primary and metastatic colon tumor samples. (E) Expression of GCC in grade 2 metastatic colorectal tumor. (F) Expression of GCC in poorly differentiated metastatic colon tumor. GCC, guanylyl cyclase C; mCRC, metastatic colorectal cancers.

GCC is an intestinal epithelial-selective gene, as demonstrated in a panel of human tissues that was assessed by quantitative RT-PCR (Fig 1A). Analysis of patient-derived tumor samples demonstrated that GCC was detected at the mRNA level in all primary and metastatic CRC tumor samples analyzed (Fig 1B). Using a custom-made GCC-specific rabbit monoclonal antibody, GCC protein expression was assessed in a repository of >300 human CRC tumor samples and normal colon samples by immunohistochemistry. GCC was found to be restricted to the apical surface of normal intestinal epithelial cells facing the lumen (Fig 1C). The staining of primary and metastatic CRC tumor samples demonstrated that GCC was expressed at a score of 50 or more (out of 600) in more than 95% of tumor samples (Fig 1D). The expression pattern fell into two distinct categories; moderately and well-differentiated tumors expressed GCC on an apical surface, while poorly differentiated tumors typically demonstrated a diffuse cytoplasmic staining pattern with varying intensity (Fig 1E and 1F). The restricted expression of GCC at the basement membrane and the tight junctions between intestinal epithelial cells could limit systemically delivered GCC-targeting agents from reaching the gut [24, 35]. In tumor cells, these interactions are disrupted, allowing free access to the antigen, making GCC one of the most tumor-selective cell surface targets to date, and thus a suitable target for ADC delivery.

### TAK-264 selectively binds, is internalized by, and is cytotoxic to GCC-expressing cells

**Fig 2:**
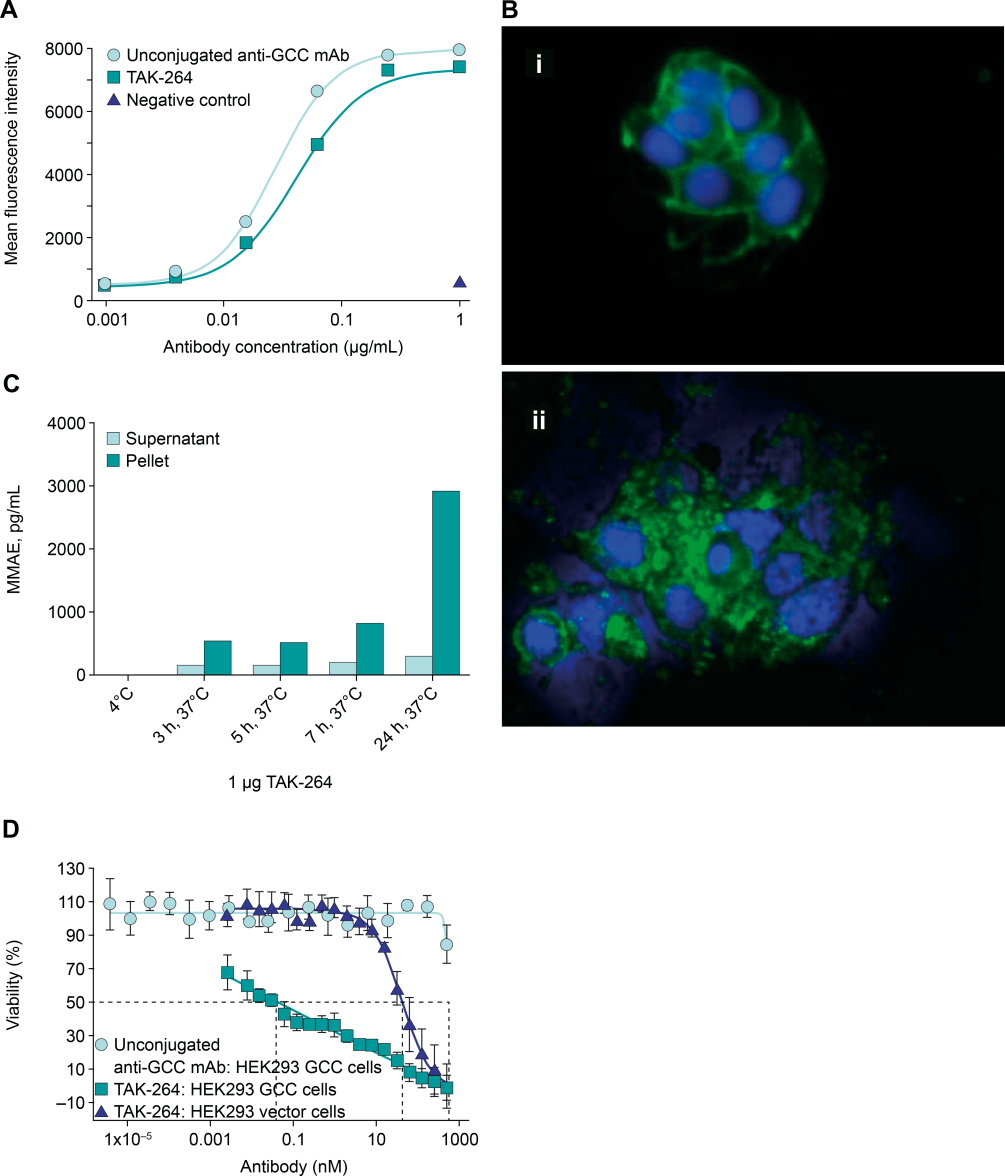
Characterization of TAK-264 binding, internalization, and *in vitro* cytotoxicity. (A) Unconjugated anti-GCC monoclonal antibody and TAK-264 bind to GCC-expressing cells. Flow cytometry was performed with unconjugated anti-GCC monoclonal antibody and TAK-264 on HEK293-GCC or HEK293-vector cells. Mean fluorescence intensity is plotted versus monoclonal antibody concentration ranging from 0.001–1 μg/mL. (B) TAK-264 is internalized by GCC-expressing cells. Internalization assays were performed with TAK-264 on HEK-GCC cells. Antibodies were incubated with cells at (i) 4°C for 30 min or at (ii) 37°C for 3 h, prior to fixing and staining with secondary antibody. Immunofluorescence was visualized with a confocal microscope (Zeiss Pascal) and analyzed using Axiovert software. (C) Time-dependent accumulation of MMAE in samples incubated with 1 μg TAK-264 at 37°C, 5% CO_2_, and analyzed by LC/MS/MS. (D) *In vitro* cytotoxicity of TAK-264. Cell viability was assessed 4 days after incubation of HEK293-GCC cells or HEK293-vector cells with increasing concentrations of unconjugated and MMAE-conjugated anti-GCC monoclonal antibody. The average of three independent experiments is plotted with error represented as SEM. GCC, guanylyl cyclase C; LC/MS/MS, liquid chromatography–tandem mass spectrometry; MMAE, monomethyl auristatin E; SEM, standard error of the mean.

TAK-264 consists of a fully human anti-GCC monoclonal antibody conjugated to MMAE via the protease-cleavable vc peptide linker [36]. Unconjugated anti-GCC monoclonal antibody and TAK-264 were compared for relative binding to HEK293-GCC, an engineered cell line that was selected for its high level of antigen expression (Fig 2A). TAK-264 and unconjugated anti-GCC monoclonal antibody bound to HEK293-GCC cells in a dose-dependent manner with similar affinities (EC_50_: 42 ng/mL and 25 ng/mL, respectively), indicating that conjugation of MMAE to the anti-GCC monoclonal antibody did not significantly affect antigen-binding properties.

Target-dependent internalization of the unconjugated anti-GCC monoclonal antibody and TAK-264 was demonstrated in HEK293-GCC cells using immunofluorescence microscopy with a fluorescently labeled anti-human IgG secondary antibody. When incubated on ice, TAK-264 was strictly localized on the cell surface (Fig 2B, top). Upon incubation at 37°C, punctuate staining was detected intracellularly as early as 15 min (Fig 2B, bottom), indicative of internalization of TAK-264. Unconjugated anti-GCC monoclonal antibody behaved identically to TAK-264 on HEK293-GCC cells (data not shown). No binding or internalization was detected for TAK-264 or the unconjugated anti-GCC monoclonal antibody with HEK293-vector control cells (data not shown). Following binding to GCC, TAK-264 was endocytosed and trafficked to the lysosomes, where MMAE is released by lysosomal proteases [37]. A time-dependent accumulation of MMAE was observed by LC/MS/MS analysis of cell culture supernatant and pellets collected from samples incubated with 1 μg TAK-264 at 37°C, 5% CO_2_ (Fig 2C).

To evaluate TAK-264 *in vitro* cytotoxicity, HEK293-GCC or HEK293-vector cells were incubated with TAK-264 or unconjugated anti-GCC monoclonal antibody alone (Fig 2D). Unconjugated anti-GCC monoclonal antibody had no effect on HEK293-GCC cell viability. However, TAK-264 potently reduced HEK293-GCC cell viability, with a 50% lethal dose (LD_50_) of 39 pM, approximately 1000-fold lower than the TAK-264 LD_50_ on HEK293-vector cells (42.5 nM), indicating that the cytotoxic activity of TAK-264 is highly target-dependent. The HEK293-GCC and HEK293-vector cells showed similar sensitivity to free MMAE (S1 Table). The cytotoxic activity of TAK-264 was substantially reduced in HT-29 cells engineered to express GCC. Interestingly, this cell line expresses less GCC at the cell surface (S2 Table), indicating that TAK-264 potency is driven at least in part by antigen surface density. We next tested the activity of TAK-264 in GCC-expressing tumor xenografts.

### *In vivo* pharmacodynamics and pharmacokinetics of TAK-264

**Fig 3:**
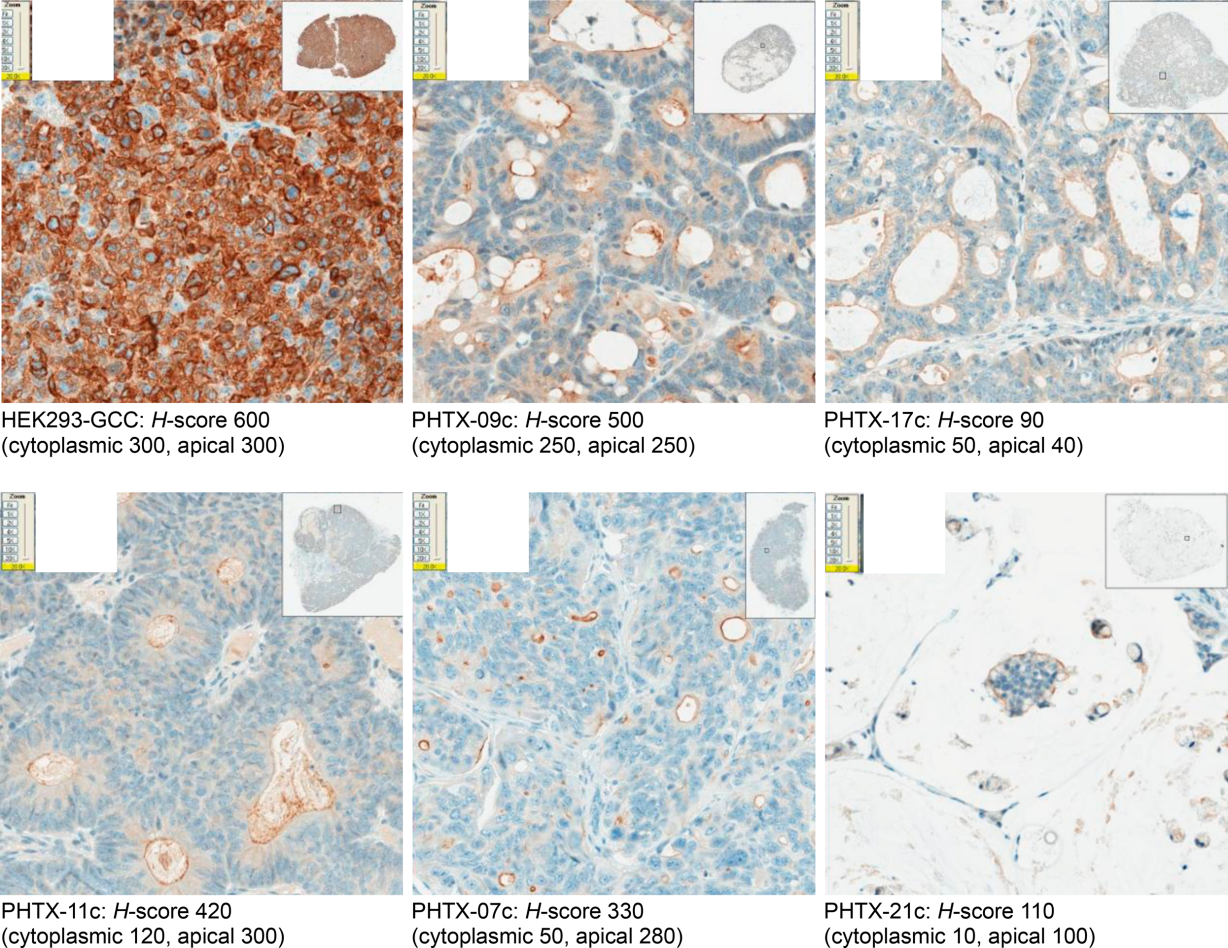
GCC protein expression in HEK293-GCC and PHTX xenograft tumors (from mCRC patients) in SCID mice. GCC, guanylyl cyclase C.

The anti-tumor activity of TAK-264 was evaluated in engineered HEK293-GCC xenograft tumors as well as in primary human tumor xenografts that had GCC expression patterns closely resembling those found in patient tumor samples. The expression of GCC was validated by immunohistochemistry. GCC levels differed among these tumors (Table 1; Fig 3). In moderate/well-differentiated tumors that maintained a polarized epithelial cellular structure, GCC was concentrated on the luminal side of the tumor tissue.

**Table 1:** Summary of TAK-264 anti-tumor activity in *in vivo* models of GCC-expressing tumor xenografts.

Model	GCC expression H-score		TAK-264 dose*	T/C at 7.5 mg/kg	P-value	T/C at 10 mg/kg	P-value
	Cytoplasmic	Apical					
HEK293-GCC	300	300	1.875–7.5 mg/kg	0.03	<0.001	NA	NA
PHTX-09c	250	250	0.938–10 mg/kg	0.16	<0.001	0.09	<0.001
PHTX-21c^†^	10	100	3.75–10 mg/kg	0.38	<0.001	0.23	<0.001
PHTX-07c^†^	50	280	3.75–10 mg/kg	0.74	<0.05	0.53	<0.001
PHTX-17c^†^	50	40	0.938–10 mg/kg	0.5	<0.001	0.46	<0.05
PHTX-11c^†^	120	300	7.5 mg/kg	0.63	<0.001	NA	NA

*Once-weekly schedule, three doses of TAK-264

^†^Included in S1 Fig.

IHC, immunohistochemistry; GCC, guanylyl cyclase C; NA, not applicable; T/C, ratio of tumor size relative to control, calculated on the last day on which control mice remained on study.

**Fig 4:**
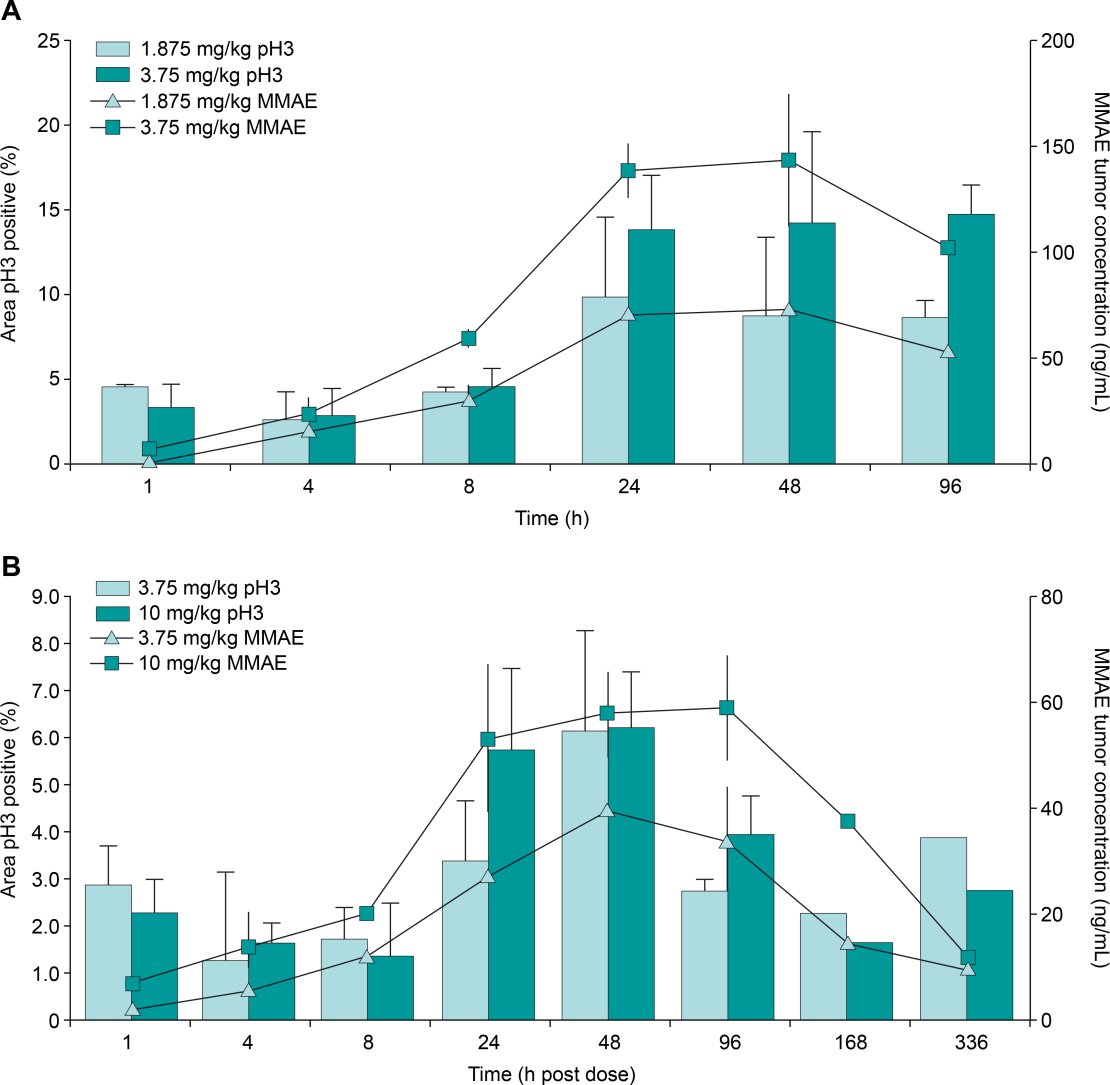
Phospho-histone H3 and MMAE levels in HEK293-GCC and PHTX-09c xenograft tumors following TAK-264 treatment. (A) HEK293-GCC and (B) PHTX-09 xenograft tumors were harvested at the indicated times post-treatment, with three animals per time point. Control tumors were taken from three untreated mice. The average percentage of phospho-histone H3-positive cells and average level of MMAE tumor concentration are plotted, with error represented as standard deviation. GCC, guanylyl cyclase C; MMAE, monomethyl auristatin E.

In order to study the relationship between uptake of antibody, release of MMAE, and the pharmacodynamic effect of TAK-264 in xenograft tumors, we measured the fraction of cells in mitosis, the concentration of free MMAE, and the uptake of radiolabeled monoclonal antibody at matched time points. Mice carrying HEK293-GCC or primary human tumor explant (PHTX)-09c tumors were evaluated by immunofluorescence for phospho-histone H3 levels, a mitotic marker, following a single IV dose of TAK-264 at 1.875 or 3.75 mg/kg. There was a time-dependent effect of TAK-264 on the tumor cells (Fig 4) that was consistent with the mechanism of action of MMAE [29, 36]. The fraction of phospho-histone H3-positive cells increased 3- to 4-fold, peaking 3–4 days post-injection. The peak concentration of MMAE delivered to the tumors by TAK-264 coincided with the peak in phospho-histone H3 staining, demonstrating a direct correlation between free MMAE and mitotic arrest. The time to peak MMAE concentrations coincided with peak antibody uptake in the tumors as assessed by single photon emission computed tomography *in vivo* imaging of ^111^In-labeled TAK-264 [35].

Hence, there was a correlation between antibody uptake in the tumor, release of MMAE from the ADC, and the effect on the tumor. HEK293-GCC tumors, which show greater GCC expression than PHTX-09c tumors, showed higher concentrations of free MMAE compared with the PHTX-09c tumors, demonstrating the target density-dependent accumulation of cytotoxic payload (S2 Fig).

### *In vivo* activity of TAK-264 in HEK293-GCC tumor-bearing mice and in PHTX models of mCRC

**Fig 5:**
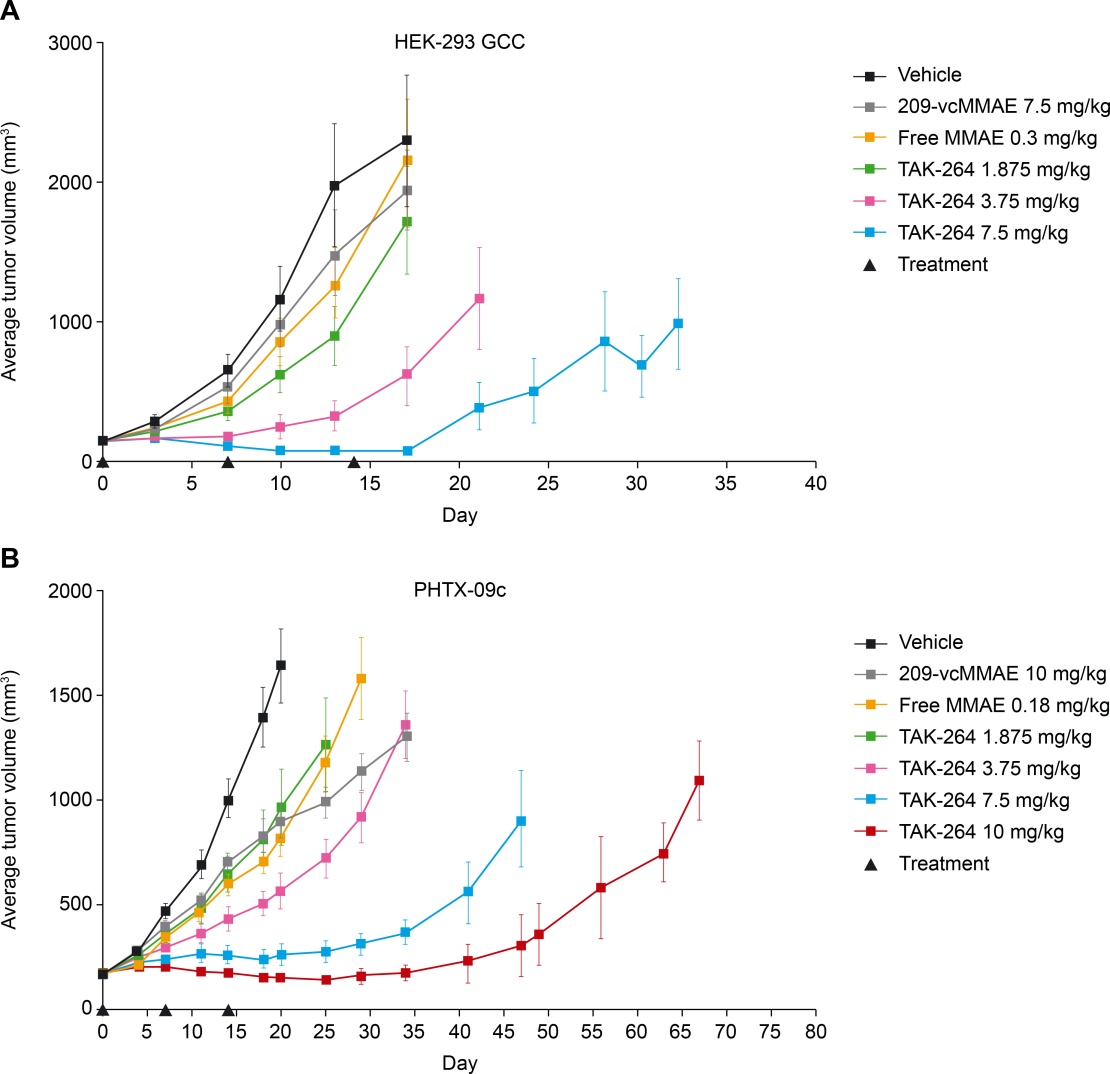
TAK-264 induces tumor regression and long-term tumor growth delay of GCC-expressing tumors. Female SCID mice bearing (A) GCC-expressing HEK293 xenograft tumors and (B) PHTX-09c tumors, a patient-derived xenograft model of mCRC, were treated when the tumor reached approximately 200 mm^3^ with either vehicle, non-targeting control antibody 209-vcMMAE, free MMAE, or TAK-264 at various doses on a weekly dosing schedule indicated by the black triangles. Average tumor volume was determined at multiple time points following start of treatment and is shown ± S.E.M.

Tumor-bearing mice were treated on a once-weekly schedule, for three doses, with vehicle, non-targeting control antibody 209-vcMMAE, free MMAE, or TAK-264 at various doses. In HEK293-GCC tumors (GCC expression H-score of 600 by immunohistochemistry; Fig 5A), treatment with non-targeting ADC (209-vcMMAE) or free MMAE alone had no effect on tumor growth; however, treatment with TAK-264 at 1.875, 3.75, or 7.5 mg/kg resulted in tumor growth inhibition in a dose-dependent fashion; respective TGI values on day 17 (last day control mice remained on study) were 55%, 84%, and 97% (P > 0.05, P < 0.01, and *P* < 0.001, respectively). In the PHTX-09c tumor model (GCC expression H-score of 500 by immunohistochemistry), dose-dependent anti-tumor activity was also observed with TAK-264 (Fig 5B); TGI values following treatment with TAK-264 at 3.75, 7.5, and 10 mg/kg were 66%, 84%, and 91%, respectively (all *P* ≤ 0.01), on day 20. TAK-264 caused regressions of tumor growth and similar activity in both models although a higher dose was required in PHTX-09c versus HEK293-GCC tumors (10 vs. 7.5 mg/kg).

TAK-264 also demonstrated TGI in other PHTX models (Table 1). Overall, TGI appeared to correlate with antigen density, with tumors with higher GCC expression generally showing greater sensitivity to TAK-264 compared to tumors with lower antigen expression levels. Of particular interest, some models were less sensitive to TAK-264 treatment even though they expressed significant levels of antigen. TAK-264 could be detected at GCC-expressing sites 7 days post-administration of TAK-264, suggesting that the ADC was able to reach GCC-expressing tumor cells (S3 Fig). Furthermore, it is not likely due to a failure of cells to internalize and/or process TAK-264 as levels of free MMAE detected in PHTX-11c, a resistant model, were similar to levels found in the PHTX-09c model following the same dose (data not shown). Drug-efflux pump expression has also been investigated by transcriptional profiling, as MMAE has been demonstrated to be a substrate, particularly for P-glycoprotein [38]; however, no significant differences could be found at the mRNA level between sensitive and resistant PHTX models (data not shown). Thus, these tumors appear inherently resistant to a high local MMAE concentration achieved with TAK-264. The mechanism of MMAE resistance is an ongoing area of investigation.

## Discussion

We have herein described the development of an ADC targeted to GCC, which is expressed in >95% of primary and metastatic CRC tumors and in 60–70% of gastric, esophageal, and pancreatic cancers [16, 17, 39]. TAK-264 consists of a fully human anti-GCC monoclonal antibody linked to the highly potent cytotoxic agent MMAE via a protease-cleavable linker (vc; MMAE and the linker technology are licensed from Seattle Genetics, Inc.). TAK-264 retained cell surface binding specificity and affinity comparable to unconjugated anti-GCC monoclonal antibody and was internalized by GCC-expressing cells. TAK-264 demonstrated potent cytotoxicity against HEK293-GCC but not HEK293-vector cells. When administered to mice bearing GCC-expressing xenografts, which had comparable levels of GCC expression to those observed in human mCRC tissue samples, a single administration of TAK-264 resulted in mitotic arrest from 24 h to 4 days post-injection. This cytotoxicity corresponded to elevated free MMAE levels in tumor tissue, indicating that proteolytic cleavage of TAK-264 occurred following internalization. TAK-264 demonstrated a dose-dependent anti-tumor activity in HEK293-GCC and human primary tumor mouse xenograft models. These data support the clinical development of TAK-264 for the treatment of GCC-expressing tumors.

Pharmacokinetic and pharmacodynamic analyses in HEK293-GCC xenograft mice indicate that TAK-264 is selectively cleaved in GCC-expressing tumor cells, leading to cell death. TAK-264 appears relatively stable in circulation, with limited release of free MMAE. These observations are consistent with previous studies that have demonstrated high serum stability and efficient targeted hydrolysis of ADCs with protease-cleavable vc-dipeptide linkers [40]. HEK293-GCC xenograft mice tolerated the administered doses of TAK-264 with the schedule tested. Doses of TAK-264 up to 7.5–10 mg/kg on a once-every-3-weeks schedule were required for an anti-tumor effect in mice. This dosing regimen was well-tolerated in all preclinical studies; however, clinical data suggests that this exceeds clinically achievable concentrations. During clinical evaluation of TAK-264, a maximum tolerated dose was determined to be 1.8 mg/kg on a once-every-3-weeks schedule [41], consistent with the maximum tolerated dose for other ADCs with the same linker payload on the same schedule [42, 43].

Many properties of GCC make it an attractive target for the development of an antibody-based therapeutic for mCRC and other GCC-expressing malignancies. The expression pattern is its foremost attribute, with expression restricted to the luminal membrane of normal intestinal tissues (large intestine, small intestine, and rectum) [10, 11] and maintained in primary CRC and mCRC tumors [12–14]. The apical-located GCC in normal intestinal epithelial cells is not in direct contact with the bloodstream [10, 24, 44], and therefore is predicted to be inaccessible by an ADC administered intravenously. In contrast, GCC expression on CRC metastases to the liver becomes less polarized and the tissue structure/vascularity within tumors could allow anti-GCC ADCs to specifically target tumor metastases while sparing normal gastrointestinal tissues. As the unconjugated anti-GCC monoclonal antibody does not cross-react with mouse GCC protein (data not shown), we were not able to test this hypothesis directly. However, rates of gastrointestinal toxicities were generally limited in the first-in-human phase 1 study, with 51% of patients reporting nausea, 49% decreased appetite, 39% diarrhea, and 24% constipation and vomiting; toxicities were generally mild or moderate in severity, with only 7% grade ≥3 diarrhea [41].

GCC expression in primary and metastatic colon tumor tissues is heterogeneous among patients, as well as within a single tumor. We chose to study TAK-264 activity in PHTX models that more closely resemble human disease and the observed GCC expression pattern. We also assumed that the range of drug levels evaluated in our preclinical experiments would be informative in the context of clinical studies, with the aim of achieving similar antitumor effects as observed in our xenograft models, i.e., TAK-264 would be expected to induce tumor cell death and reduce tumor burden in mCRC patients and other GCC-expressing cancers. Our *in vivo* experiments suggest that TAK-264 cytotoxic activity may be dependent on antigen expression density, as assessed by immunohistochemistry. Determining GCC expression level and how it relates to antitumor activity will be an objective during the clinical development of TAK-264. Furthermore, it will be important to define non-invasive biomarkers to select the appropriate responsive patient population.

Observations in PHTX models suggest that some tumors may be refractory to TAK-264 despite moderate to high GCC expression, possibly suggesting a degree of TAK-264 resistance. The defined mechanism of TAK-264 resistance is an area of ongoing investigation. A study has shown that MMAE is a substrate for some drug pumps [45]. It is possible that over-expression of pumps may render tumors less sensitive to the effects of TAK-264. The spindle checkpoint is an evolutionary conserved mechanism involving several key regulators that monitor fidelity of chromosome separation during mitosis [46]. A number of *in vitro* studies have indicated that dysregulation of key spindle checkpoint regulators compromise the sensitivity of cancer cells to microtubule inhibitors such as paclitaxel and docetaxel [47–50]. This may explain why these agents have failed to demonstrate significant clinical benefit in CRC [46]. Indeed, 80–85% of CRC tumors display chromosomal instability that can sometimes be attributable to expression abnormalities or somatic mutations in spindle checkpoint regulators [51]. In clinical studies, the cancer molecular subtype (chromosomal instability, microsatellite instability phenotype, and CpG island methylator phenotype) and expression levels/mutation status of various genes could be assessed to help identify biomarkers that could stratify patient populations likely to respond to TAK-264.

In summary, we have demonstrated that TAK-264 is cytotoxic *in vitro* and *in vivo* to GCC-expressing cells, providing a solid scientific rationale for further preclinical and clinical development of this drug. As GCC is a highly specific colon epithelial antigen that is expressed on both primary and metastatic CRC tumors as well as gastric, esophageal, and pancreatic tumors, TAK-264 may provide a novel targeted approach for the treatment of these GCC-expressing malignancies, which are diseases of unmet medical need.

## Supporting information

S1 Table *In vitro* cytotoxicity of TAK-264 and free MMAE in HEK293-GCC and HEK293-vector cells and in HT29-GCC and HT29-vector cells. HT29-GCC cells were engineered to express GCC but with fewer GCC molecules per cell compared with HEK293-GCC.
(DOCX)

S2 Table Relative cell surface expression of GCC in HEK293-GCC and HT29-GCC cells, as measured by fluorescence activated cell sorting (mean fluorescence intensity normalized to control). (DOCX)

S1 Fig TAK-264 activity in GCC-positive PHTX models of colorectal cancer. (TIF)

S2 Fig Pharmacokinetic and pharmacodynamic characteristics of TAK-264 in HEK293-GCC or PHTX-09c tumor-bearing SCID mice. (TIF)

S3 Fig TAK-264 human Ig chain in 7 days post-administration PHTX-09c xenografts localizes to sites of GCC expression in serial sections. (TIF)

S1 File NC3Rs checklist Page 1. (PDF)

S2 File NC3Rs checklist Page 2. (PDF)
