# Supplementary material for: A monomethyl auristatin E-conjugated antibody to guanylyl cyclase C is cytotoxic to target-expressing cells in vitro and in vivo
Source: PLoS One. 2018 Jan 25;13(1):e0191046. doi: 10.1371/journal.pone.0191046 (PMC5784926; doi:10.1371/journal.pone.0191046)
Supplement: S1 Table — HT29-GCC cells were engineered to express GCC but with fewer GCC molecules per cell compared with HEK293-GCC. (DOCX) [file pone.0191046.s001.docx]

**Supplementary Table S1. *In vitro* cytotoxicity of TAK-264 and free MMAE in HEK293-GCC and HEK293-vector cells and in HT29-GCC and HT29-vector cells. HT29-GCC cells were engineered to express GCC but with fewer GCC molecules per cell compared with HEK293-GCC.**

| **Cells** | **TAK-264, nM** | | **Free MMAE, nM** | |
| --- | --- | --- | --- | --- |
|  | **LD_50_** | **SD** | **LD_50_** | **SD** |
| HEK293-GCC | 0.192 | 0.005 | 0.07 | 0.06 |
| HEK-293 vector | 106 | 0.021 | 0.04 | 0.03 |
| HT29-GCC | 17.1 | 0.02 | 0.04 | 0.01 |
| HT29-vector | 71.8 | 0.03 | 0.07 | 0.04 |

GCC, guanylyl cyclase C; LD_50_, 50% lethal dose; MMAE, monomethyl auristatin E; SD, standard deviation.
