# Supplementary material for: A monomethyl auristatin E-conjugated antibody to guanylyl cyclase C is cytotoxic to target-expressing cells in vitro and in vivo
Source: PLoS One. 2018 Jan 25;13(1):e0191046. doi: 10.1371/journal.pone.0191046 (PMC5784926; doi:10.1371/journal.pone.0191046)
Supplement: S2 Table — (DOCX) [file pone.0191046.s002.docx]

**Supplementary Table S2. Relative cell surface expression of GCC in HEK293-GCC and HT29-GCC cells, as measured by fluorescence activated cell sorting (mean fluorescence intensity normalized to control).**

| **Relative fluorescence** | **HEK293-GCC** | **HT29-GCC** |
| --- | --- | --- |
| Secondary PE-conjugated detection antibody only | 134 | 162 |
| With 400 ng/mL of anti-GCC primary antibody plus secondary detection antibody | 5842 | 1176 |
| Fold-change versus control | 43.6 | 7.3 |

GCC, guanylyl cyclase C.
