# Supplementary figures and images for: A monomethyl auristatin E-conjugated antibody to guanylyl cyclase C is cytotoxic to target-expressing cells in vitro and in vivo
Source: PLoS One. 2018 Jan 25;13(1):e0191046. doi: 10.1371/journal.pone.0191046 (PMC5784926; doi:10.1371/journal.pone.0191046)

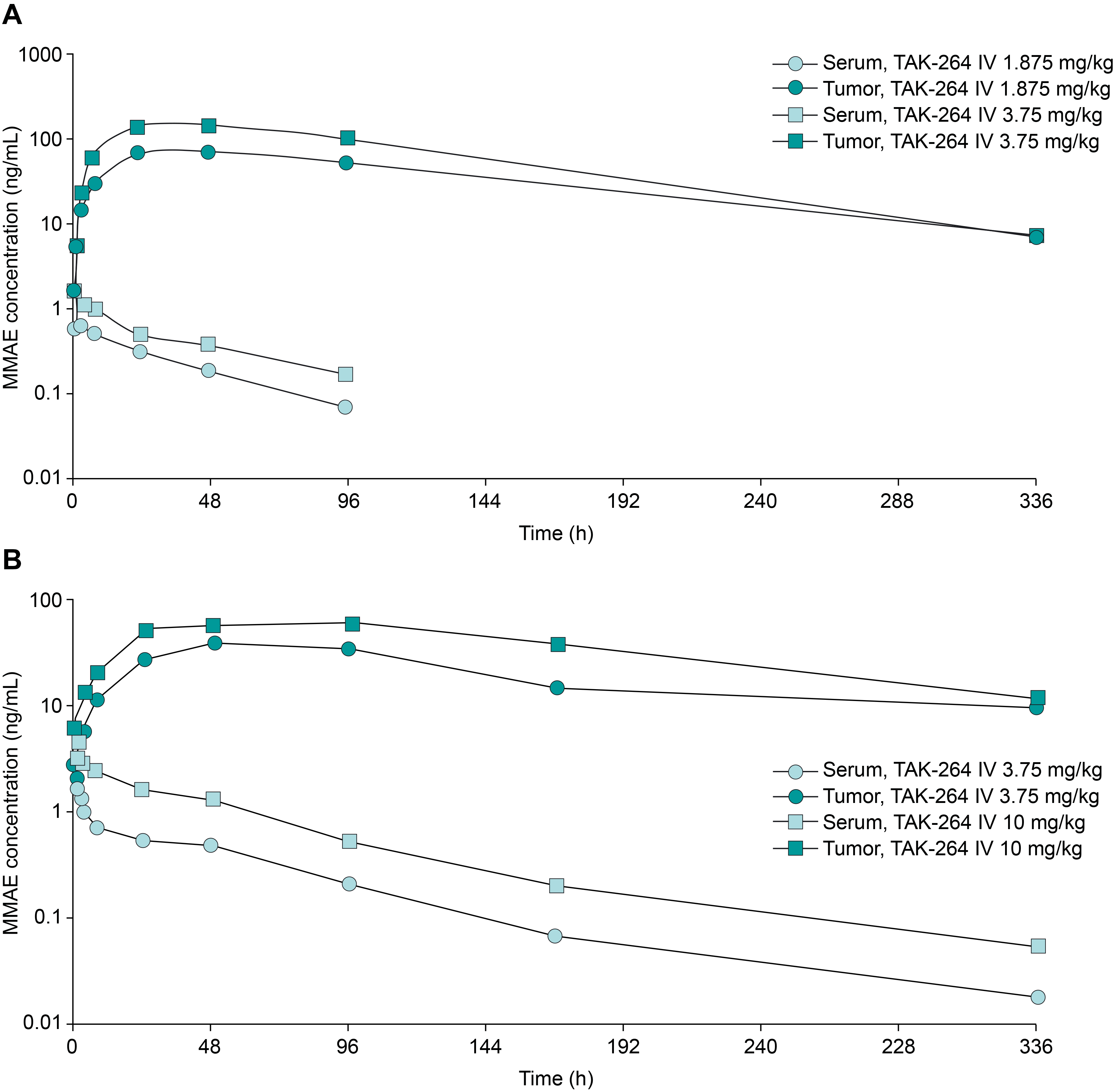

Supplement: S2 Fig — (TIF) [file pone.0191046.s004.tif]

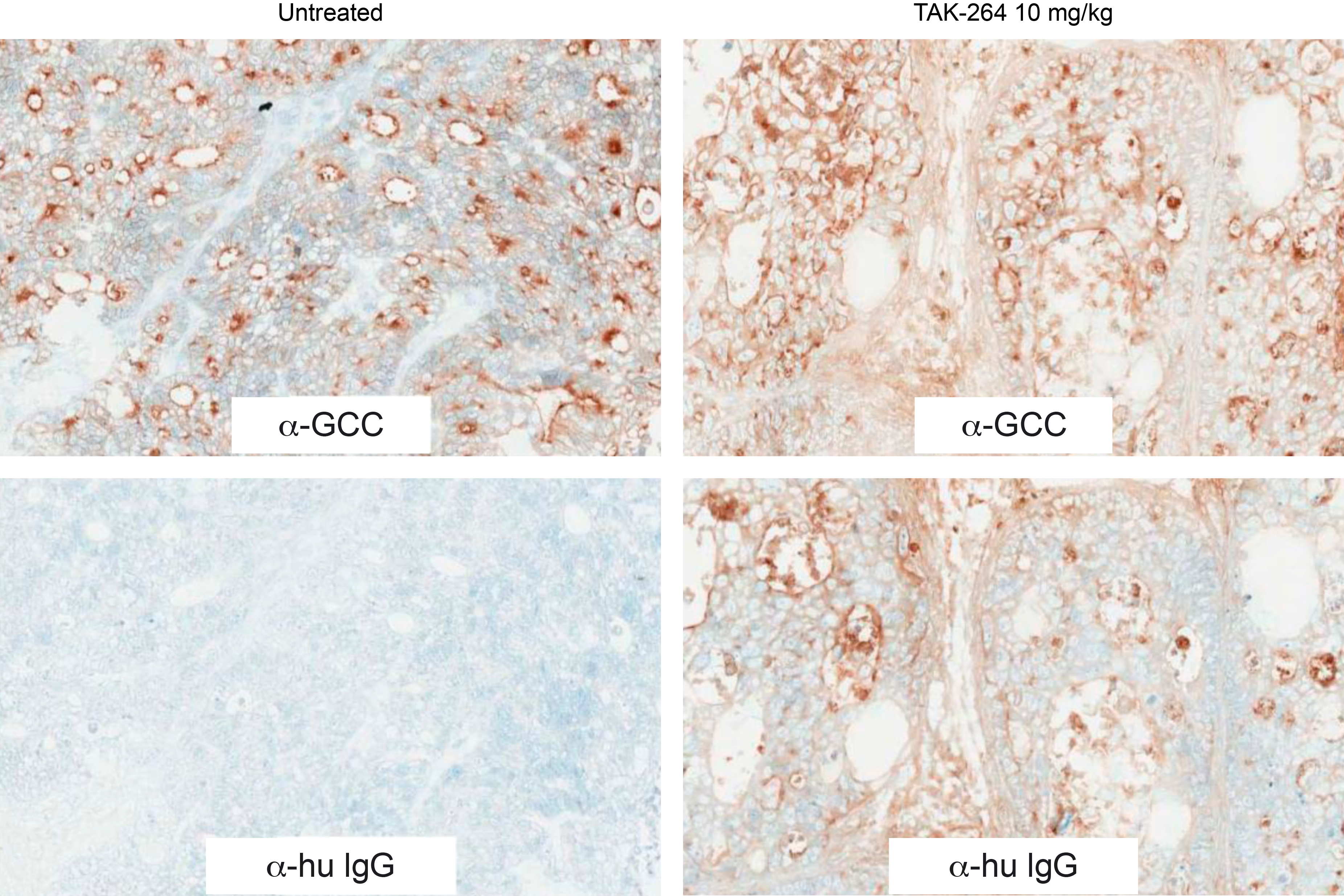

Supplement: S3 Fig — (TIF) [file pone.0191046.s005.tif]
