# Supplementary material for: A monomethyl auristatin E-conjugated antibody to guanylyl cyclase C is cytotoxic to target-expressing cells in vitro and in vivo
Source: PLoS One. 2018 Jan 25;13(1):e0191046. doi: 10.1371/journal.pone.0191046 (PMC5784926; doi:10.1371/journal.pone.0191046)
Supplement: S2 File — (PDF) [file pone.0191046.s007.pdf]

Housing and husbandry	9	Provide details of:<br>a. Housing (type of facility e.g. specific pathogen free [SPF]; type of cage or housing; bedding material; number of cage companions; tank shape and material etc. for fish<br>b. Husbandry conditions (e.g. breeding programme, light/dark cycle, temperature, quality of water etc for fish, type of food, access to food and water, environmental enrichment).<br>c. Welfare-related assessments and interventions that were carried out prior to, during, or after the experiment.	a. - c. Human xenograft tumor studies/ Paragraph 1 & 5
Sample size	10	a. Specify the total number of animals used in each experiment, and the number of animals in each experimental group.<br>b. Explain how the number of animals was arrived at. Provide details of any sample size calculation used.<br>c. Indicate the number of independent replications of each experiment, if relevant.	a. Human xenograft tumor studies/ Paragraphs 3 & 4<br>b. not in manuscript<br>c. not relevant
Allocating animals to experimental groups	11	a. Give full details of how animals were allocated to experimental groups, including randomisation or matching if done.<br>b. Describe the order in which the animals in the different experimental groups were treated and assessed.	a. & b. Human xenograft tumor studies/ Paragraphs 3 & 4
Experimental outcomes	12	Clearly define the primary and secondary experimental outcomes assessed (e.g. cell death, molecular markers, behavioural changes).	Abstract, Introduction/ Paragraph 5, Discussion/ Paragraph 2 & 4
Statistical methods	13	a. Provide details of the statistical methods used for each analysis.<br>b. Specify the unit of analysis for each data set (e.g. single animal, group of animals, single neuron).<br>c. Describe any methods used to assess whether the data met the assumptions of the statistical approach.	Statistical Analysis
RESULTS			
Baseline data	14	For each experimental group, report relevant characteristics and health status of animals (e.g. weight, microbiological status, and drug or test naïve) prior to treatment or testing. (This information can often be tabulated).	Human xenograft tumor studies/Paragraph 5
Numbers analysed	15	a. Report the number of animals in each group included in each analysis. Report absolute numbers (e.g. 10/20, not 50% [2]).<br>b. If any animals or data were not included in the analysis, explain why.	Human xenograft tumor studies/Paragraphs 3 & 4
Outcomes and estimation	16	Report the results for each analysis carried out, with a measure of precision (e.g. standard error or confidence interval).	Statistical Analysis, Figure 1 description
Adverse events	17	a. Give details of all important adverse events in each experimental group.<br>b. Describe any modifications to the experimental protocols made to reduce adverse events.	Human xenograft tumor studies/Paragraph 5
DISCUSSION			
Interpretation/ scientific implications	18	a. Interpret the results, taking into account the study objectives and hypotheses, current theory and other relevant studies in the literature.<br>b. Comment on the study limitations including any potential sources of bias, any limitations of the animal model, and the imprecision associated with the results[2]. .<br>c. Describe any implications of your experimental methods or findings for the replacement, refinement or reduction (the 3Rs) of the use of animals in research.	a. Throughout<br>b. N/A<br>c. N/A
Generalisability/ translation	19	Comment on whether, and how, the findings of this study are likely to translate to other species or systems, including any relevance to human biology.	Abstract, Introduction/ Paragraph 2, Results/ Paragraph 1, Figure 1 description, Discussion/Paragraph 1-6
Funding	20	List all funding sources (including grant number) and the role of the funder(s) in the study.	N/A - Privately funded. Takeda Corp.

References:

1. Kilkenny C, Browne WJ, Cuthill IC, Emerson M, Altman DG (2010) Improving Bioscience Research Reporting: The ARRIVE Guidelines for Reporting Animal Research. PLoS Biol 8(6): e1000412. doi:10.1371/journal.pbio.1000412
2. Schulz KF, Altman DG, Moher D, the CONSORT Group (2010) CONSORT 2010 Statement: updated guidelines for reporting parallel group randomised trials. BMJ 340:c332.
